# Translation to Brazilian Portuguese, cultural adaptation and reproducibility of the questionnaire “Ankylosing Spondylitis: What do you know?” Tradução para a língua portuguesa brasileira, adaptação cultural e reprodutibilidade do questionário “Ankylosing Spondylitis: What do you know?”

**DOI:** 10.1590/1516-3180.2016.0084310516

**Published:** 2016-09-26

**Authors:** Aline Orlandi, Christine Brumini, Anamaria Jones, Jamil Natour

**Affiliations:** I PT. Postgraduate Student, Rheumatology Division, Escola Paulista de Medicina, Universidade Federal de São Paulo (EPM-Unifesp), São Paulo, SP, Brazil.; II PT, MSc. Postgraduate Student, Rheumatology Division, Escola Paulista de Medicina, Universidade Federal de São Paulo (EPM-Unifesp), São Paulo, SP, Brazil.; III PT, PhD. Affiliate Professor, Rheumatology Division, Escola Paulista de Medicina, Universidade Federal de São Paulo (EPM-Unifesp), São Paulo, SP, Brazil.; IV MD, PhD. Associate Professor, Rheumatology Division, Escola Paulista de Medicina, Universidade Federal de São Paulo (EPM-Unifesp), São Paulo, SP, Brazil.

**Keywords:** Spondylitis, ankylosing, Knowledge, Translating, Education, Surveys and questionnaires, Espondilite anquilosante, Conhecimento, Tradução, Educação, Inquéritos e questionários

## Abstract

**CONTEXT AND OBJECTIVE:**

Ankylosing spondylitis (AS) generates inflammation and pain in entheses, peripheral joints and the spine. Education regarding AS can improve patients’ disability. Thus, it is important to assess patients’ knowledge. There is no instrument in the literature for assessing knowledge of AS in Portuguese. The aim here was to translate to the Brazilian Portuguese language, culturally adapt and test the reliability of the questionnaire “Ankylosing Spondylitis: What do you know?” and to correlate the findings with other factors.

**DESIGN AND SETTING:**

Original article regarding validation of questionnaire, produced at the Federal University of Sao Paulo (Unifesp).

**METHODS:**

For translation and cultural adaptation, Guilleman methodology was used. After the first phase, the reliability was tested on 30 patients. Correlations between these scores and other factors were examined.

**RESULTS:**

In the interobserver assessment, the Pearson correlation coefficient and Cronbach’s alpha were 0.831 and 0.895, respectively. In the intraobserver evaluation, the intraclass correlation coefficient and Cronbach’s alpha were 0.79 and 0.883, respectively. At this stage, the score for area of knowledge A showed correlations with ethnicity and education; the score for area D, with age; the total score and scores for areas A and B with “social aspects” of SF-36; and the score for area D with “pain”, “vitality” and “emotional aspects” of SF-36.

**CONCLUSION:**

The Brazilian version of the questionnaire “Ankylosing Spondylitis: What do you know?” was created. It is reproducible and correlates with education level, ethnicity and the SF-36 domains “social aspects” and “emotional aspects”.

## RESUMO

**CONTEXTO E OBJETIVO:**

A espondilite anquilosante (EA) gera inflamação e dor em enteses, articulações periféricas e coluna vertebral. A educação na EA pode melhorar a incapacidade dos pacientes. Assim, é importante avaliar o conhecimento do paciente. Não há na literatura instrumento que avalie o conhecimento da EA na língua portuguesa. O objetivo foi traduzir para a língua portuguesa brasileira, realizar a adaptação cultural e testar a confiabilidade do questionário Ankylosing Spondylitis: What do you know? e relacionar os resultados com outros fatores.

**TIPO DE ESTUDO E LOCAL:**

Artigo original de validação de questionário realizado na Universidade Federal de São Paulo (Unifesp).

**MÉTODOS:**

Para tradução e adaptação cultural, foi utilizada a metodologia de Guilleman. Depois da primeira fase, a confiabilidade foi testada em 30 pacientes. Correlações entre esses escores e outros fatores foram avaliadas.

**RESULTADOS:**

Na avaliação interobservador, o coeficiente de correlação de Pearson e o alpha de Cronbach foram de 0,831 e 0,895, respectivamente. Na avaliação intra-observador, o coeficiente de correlação intraclasse e o alpha de Cronbach foram de 0,79 e 0,883, respectivamente. Nesta etapa, foram encontradas correlações entre etnia e escolaridade e a área de conhecimento A, e entre idade e a área D; entre o escore total e escores das áreas A e B e “aspectos sociais” do SF-36 e entre a área D e “dor”, “vitalidade” e “aspectos emocionais” do SF-36.

**CONCLUSÃO:**

Foi criada a versão brasileira do questionário “Ankylosing Spondylitis: What do you know?”, que é reprodutível e se correlaciona com escolaridade, etnia e os domínios “aspectos sociais” e “aspectos emocionais” do SF-36.

## INTRODUCTION

Ankylosing spondylitis (AS) is a chronic inflammatory disease that mainly affects the spine. It may progress to morning stiffness and progressive functional limitation of the axial skeleton, and may also lead to peripheral involvement. It usually starts in young adults, mostly white males and human leucocyte antigen-B27 (HLAB27)-positive individuals.^1^

An epidemiological study conducted in all regions of Brazil showed that out of 1036 patients with spondyloarthritis, 72.3% were diagnosed with ankylosing spondylitis, 13.7% psoriatic arthritis, 6.3% undifferentiated spondyloarthropathy, 3.6% reactive arthritis, 3.1% juvenile spondyloarthropathy and 1% arthritis relating to inflammatory bowel disease. Additionally, 73.6% of the patients were men. With regard to ethnicity, 59.5% were white, 5.2% black, 20.7% mixed race and 14.6% other ethnicities, such as brown and Asian. Axial disease was observed in 36.7% and axial involvement associated with peripheral and enthesis joints in 7.9%.^2^

Like in many other rheumatic diseases, the social and medical costs are high. Indirect costs associated with days of missed work and lost productivity are the highest element of these costs, due to the large functional limitations caused by the disease.^3^

The Modified New York Criteria are most commonly used for confirmation of a diagnosis of AS. These combine clinical and radiographic criteria.^4^

Educational interventions and their implications regarding patients’ knowledge, self-efficacy, preparation and coping in relation to their illness have been studied and closely correlated with treatments for serious diseases such as cancer.^5^ Regarding multiple sclerosis, improved self-efficacy has been more closely correlated with better quality of life than has physical activity.^6^ It is believed that such relationships are also strong in connection with other diseases, such as AS.

Education for patients with inflammatory arthritis is highly recommended by the European League against Rheumatism (EULAR). This organization recently developed a consensus through analysis of the literature and experts’ opinions.^7^

One method of evaluating the efficacy of an educational program consists of measuring the modification of the patient’s knowledge.^8^ Lubrano et al. created an AS knowledge questionnaire that they named “Ankylosing Spondylitis: What do you know?” (ASWK),^9^ which was later on validated among 62 patients in the United Kingdom (UK). The questions were created by consulting four rheumatologists, a physiotherapist, an occupational therapist and two research nurses. The instrument contains 14 questions, with 72 possible answers, among which only 25 are correct. It is divided into four areas of knowledge:

1. general knowledge, etiology, symptoms and blood tests;
2. immunogenic test ­(HLA-B27) and inheritance;
3. drug treatment and physiotherapy; and
4. joint protection and energy conservation.

The final score is calculated by adding one point for each correct answer. The questionnaire was considered to be a good tool for detecting these patients’ knowledge level, while even being sensitive to small changes in theoretical knowledge levels.

The questionnaire was translated into French and it was concluded that the level of patients’ knowledge among the French population seemed to be inadequate.^10^ We believe this finding may also be true for other populations worldwide.

Therefore, while implementation of an educational program for patients affected by AS is extremely important, so is validation of its efficacy. However, in the literature, there is no questionnaire for assessing knowledge among patients with AS that has been validated for use in Portuguese.

## OBJECTIVE

The aim of this study was to translate into Portuguese, culturally adapt and test the reliability of the ASWK questionnaire. In addition, we aimed to correlate the findings regarding knowledge of the disease, i.e. pain, function, disease activity and quality of life, with personal and demographic data such as gender, age, marital status, education level, profession, disease duration, time of diagnosis and medication.

## METHODS

This study was conducted in three distinct stages. Firstly, we translated the instrument into Brazilian Portuguese, taking into consideration all the necessary cultural adaptations. Secondly, we tested the reliability of the ASWK questionnaire for the Brazilian population and, thirdly, we correlated AS knowledge levels with other parameters collected from the patients.

We interviewed 60 patients who had been diagnosed with AS in accordance with the Modified New York Criteria.^2^ These patients were of both genders and aged between 18 and 65, and they were selected at our institution’s outpatient clinics.

The study was approved by our institution’s Ethics Committee through the registration number CAAE-01752512.0.0000.5505. All the patients signed an informed consent statement confirming their agreement to participate in the study.

### Translation and cultural adaptation

After the original authors had authorized translation of the questionnaire, we started the process of translating and adapting the instrument, following the systematization proposed by Guillemin et al. and Beaton et al.^11^^,^^12^^,^^13^

### Translation into Portuguese

The translation was carried out by two English teachers whose first language was Portuguese and who did not know the original questionnaire, but were informed about the purpose of the study. The teachers worked independently and therefore produced two versions of the questionnaire in Portuguese. Later on, these were compared by a multidisciplinary team consisting of one rheumatologist and two physiotherapists. The professionals examined the two versions in order to search for any discrepancies between them. They also analyzed the applicability of each question to finally obtain a single version of the translation (V1).

### Rating of the initial translation (back translation)

V1 was then translated back into English, separately by two other English teachers whose first language was English. At this stage, the translators had no knowledge about the original questionnaire or the objectives of this study.

The two new versions were compared with the original questionnaire in order to analyze the semantic equivalence, thus allowing the V1 questionnaire to be accepted as the final version in Portuguese. The final Brazilian version is called “Espondilite anquilosante: o que você sabe a respeito?” (EAVS).

### Rating of understanding of questionnaire (cultural adaptation)

The EAVS V1 questionnaire was administered to 30 patients with AS in conformity with the Modified New York Criteria.^2^ Questions or items that were not understood by 20% or more of the patients were analyzed by the multidisciplinary group with the aim of possibly modifying them so as to maintain the original objectives of the questionnaire. All the modified questions would be applied to a new group of 30 patients to check their understanding. If necessary, the questions would again be modified until they were fully understood by 80% or more of the participants.

### Evaluation of EAVS reliability

After translation and cultural adaptation, the questionnaire was applied to a new group of 30 patients who had been diagnosed with AS, in accordance with the Modified New York Criteria,^4^ with three evaluations.

The first two evaluations were performed consecutively on the same day by two researchers (interobserver assessment). The third evaluation was carried out 7-14 days after the initial assessment by one of the previous researchers (intraobserver assessment).

### Correlation with clinical and demographic parameters

At this stage, just after the intraobserver assessment, instead of the EAVS instrument, we applied an evaluation sheet and other questionnaires in order to gather data on patients’ identification and disease characteristics. All the information collected was used to make correlations with specific AS knowledge and other parameters of the disease. We also evaluated the following:

1. pain, using the 10 cm Numerical Rating Scale for Pain,^14^ which is a 10 cm scale, numbered 0-10, that patients mark according to their level of pain;
2. functionality, using the Bath Ankylosing Spondylitis Functional Index (BASFI),^15^ which consists of 10 questions about AS patients’ functional capacity to complete daily tasks, and the Health Assessment Questionnaire for Spondyloarthropathies (HAQS),^16^ a questionnaire on daily activities in which patients are directed to choose between the responses “no difficulty”, “some difficulty”, “a great amount of difficulty” and “unable to complete task”, according to their limitations;
3. mobility, through the Bath Ankylosing Spondylitis Metrology Index (BASMI),^17^ which consists of five measurements: wall-tragus distance, lumbar flexion, cervical rotation, lateral lumbar flexion and intermalleolar distance; each measurement is awarded a score of: 0 (mild disease), 1 (moderate disease) or 2 (advanced disease), thus resulting in the final BASMI score of 0-10; and activity, through the bath ankylosing spondylitis disease activity index (BASDAI),^15^ which consists of six questions relating to five symptoms from the preceding week: tiredness, joint pain, lumbar pain, morning pain and stiffness, which were evaluated using a 10 cm horizontal visual analogue scale (VAS); and finally
4. quality of life, by applying the Short-Form-36 ­(SF-36),^18^ which evaluates the quality of life of the general population, and the Ankylosing Spondylitis Quality of Life (ASQoL),^19^ which evaluates the quality of life of patients with AS.

In addition to making correlations using the above questionnaires, the total score and the score from each survey area were correlated with the clinical parameters of the disease and with the demographic data.

### Statistical analysis

The clinical and demographic data at the cultural adaptation and reliability stages were analyzed using descriptive statistics: mean and standard deviation for categorical variables; and frequency and percentage for numerical variables.

Reliability was evaluated by means of Student’s t test. In this analysis, we used the Pearson correlation coefficient for the interobserver analysis and the intraclass correlation coefficient for the intraobserver analysis. For both analyses, we used Cronbach’s alpha coefficient to analyze the internal consistency of the instrument.

In order to correlate the results from the knowledge questionnaire with the clinical and demographic parameters of the disease, we used Student’s t test for categorical data, Pearson’s correlation coefficient for normal numerical data and Spearman’s correlation coefficient for non-normal data. To correlate the scores from the EAVS questionnaire with the data from the other questionnaires, we used Pearson’s correlation coefficient.

The significance level was set at P < 0.05. The Statistical Package for the Social Sciences (SPSS) 19.0 software was used for the analysis.

## RESULTS

V1 was reviewed and modified by the multidisciplinary committee following the two initial translations in order to ensure that the content and grammar suited the Portuguese language and Brazilian culture.

Alternatives “a” and “b” of question 14 were modified by the committee. Originally, alternative “a” stated: “Parents with ankylosing spondylitis are more likely to have children with ankylosing spondylitis”; and “b”: “Parents with ankylosing spondylitis are less likely to have children with ankylosing spondylitis”. After reaching a consensus to change them so as to better match the content, the wording became, for “a”: “Parents with ankylosing spondylitis have a great chance of having children with ankylosing spondylitis”; and “b”: “Parents with ankylosing spondylitis have little chance of having children with ankylosing spondylitis”.

### Cultural adaptation

Thirty patients who had been diagnosed with AS participated in the initial stage of implementation of the EAVS questionnaire to check its cultural equivalence. No grammatical changes to V1 were needed. The final version is shown in Appe ndix 1.

Table 1 shows the clinical and demographic data of the 30 patients diagnosed with AS who were included in the cultural adaptation phase of the Portuguese version of the questionnaire.

**Table 1.**
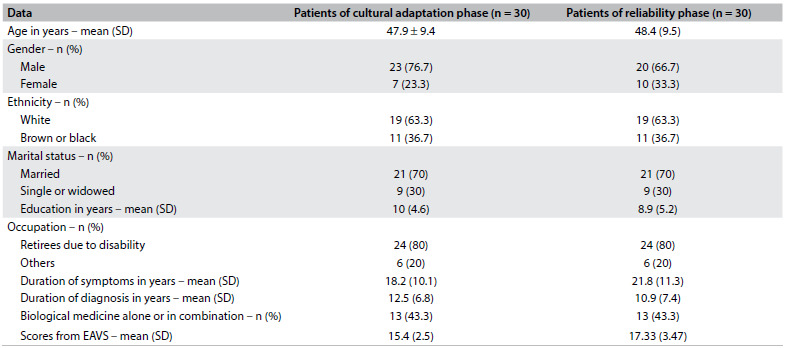
Clinical and demographic characteristics of the 30 patients included in the cultural adaptation and reliability phases

SD = standard deviation; EAVS = Questionnaire in Portuguese: “Espondilite anquilosante: o que você sabe a respeito?”

In correlating the total score from the EAVS questionnaire with the clinical and demographic data, we found correlations for education, using Spearman’s correlation coefficient (0.444, with P = 0.014); and for ethnicity, using Student’s t test (P = 0.023), such that white participants had higher scores in the questionnaire.

### Reliability

At the reliability assessment stage, the translated questionnaire was applied again to the 30 patients with AS. Table 1 shows the clinical and demographic characteristics of these patients.

In the interobserver analysis, Pearson’s correlation coefficient was r = 0.813, with P < 0.001. In the intraobserver analysis, the intraclass correlation coefficient was found to be r = 0.790, with P < 0.001. Cronbach’s alpha coefficients were 0.895 and 0.883, respectively. These data are shown in Table 2.

**Table 2.**
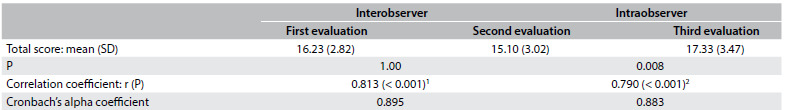
Interobserver and intraobserver reliability

SD = standard deviation; ^1^Pearson’s correlation; ^2^Intraclass correlation.

From analysis on the correlation between the total score of the questionnaire and the demographic data and clinical data on the disease, we found a correlation regarding education. Spearman’s correlation coefficient was 0.587, with P = 0.001, as shown in Table 3. No correlation was found between the total score and the clinical and categorical demographic data. These data are presented in Table 4.

**Table 3.**
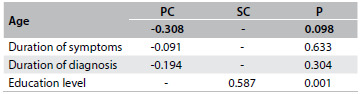
Correlation of the total score with the numerical clinical and demographic data in the reliability assessment phase

PC = Pearson’s correlation coefficient; SC = Spearman’s correlation coefficient.

**Table 4.**
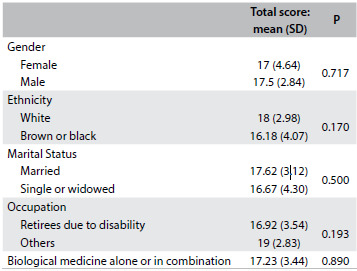
Correlation of the total score with the categorical clinical and demographic data in the reliability assessment phase

SD = standard deviation.

Finally, the total score and the scores from areas of knowledge A (“general knowledge, etiology”) and B (“immunogenic test HLA-B27”) showed negative correlations with the “social aspects” domain of SF-36. Also, the score from area D (“joint protection and energy conservation”) showed a negative correlation with the domains of “pain”, “emotional aspects” and “vitality” of SF-36. These data and all relationships are presented in Table 5.

**Table 5.**
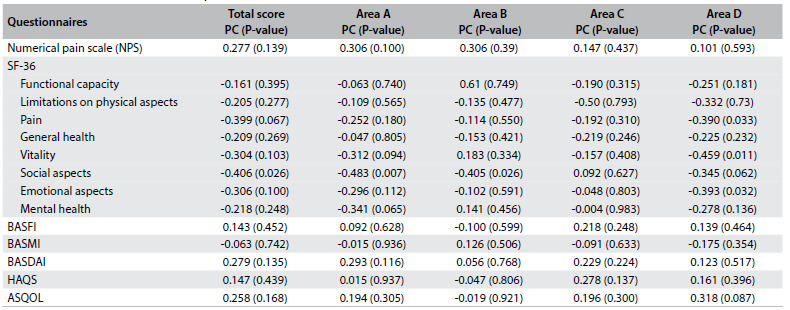
Correlations between the questionnaires

PC = Pearson’s correlation coefficient; Area A = general knowledge, etiology; Area B = immunogenic test HLA-B27; Area C = drug treatment and physiotherapy; Area D = joint protection and energy conservation; BASFI = Bath Ankylosing Spondylitis Functional Index; BASMI = Bath Ankylosing Spondylitis Metrology Index; BASDAI = bath ankylosing spondylitis disease activity index; HAQS = Health Assessment Questionnaire for Spondyloarthropathies; ASQOL = Ankylosing Spondylitis Quality of Life.

## DISCUSSION

There are a few questionnaires in Portuguese that are designed to assess patients’ knowledge of certain diseases, but none of them are specific to AS. We chose to translate the ASWK^5^ into Portuguese because it is a valid and reproducible instrument that had already been translated into another language.^10^ The ASWK translation into Portuguese was conducted by means of internationally recommended methods.^11^^,^^12^^,^^13^

In the translation phase, the multidisciplinary committee changed two questions to better match the content of the original questionnaire. During the cultural adaptation, there was no need to change any questions, since the participants did not present any issues regarding content, as had occurred in France and the United Kingdom.^9^^,^^10^

In both groups, most of the patients who were evaluated had not completed high school education. We found a strong correlation between education and the level of knowledge, in the cultural adaptation and reproducibility phase. This information is important and can explain the level of knowledge presented by the participants in this study. In the cultural adaptation phase, the average EAVS score was 15.3, i.e. lower than the scores found in France and the United Kingdom, which presented 16.4 and 19.4, respectively.^9^^,^^10^ In the reliability phase, the average was higher than in the cultural adaptation phase: 17.3.

In the UK, the patients’ level of knowledge was considered high.^9^ On the other hand, both in France and among our patients, it was low.^10^

For the British and French studies, the questionnaire was self-administered.^9^^,^^10^ However, that differs from what is done in relation to most questionnaires used in Brazil, as a consequence of the major disparity in education levels attained between these countries.

In the cultural adaptation phase, we found statistically significant differences between ethnic groups regarding knowledge, and also a correlation with education in the reliability phase. The observed low level of knowledge about AS was correlated with ethnicity and educational levels, thus reflecting the educational and racial situation in Brazil.

No correlation was found between duration of the disease and knowledge of the disease, unlike what was seen in the British study, and in other studies on individuals with rheumatoid arthritis^20^^,^^21^ and psoriatic arthritis.^22^

The questionnaire showed satisfactory levels of inter and intraobserver reliability, with r = 0.813 and r = 0.790 for the two analyses, with high correlations. It has been reported in the literature that the interobserver correlation is generally higher than the intraobserver correlation. Although the evaluations were carried out by two different interviewers, they were performed on the same day, which would explain the higher correlations in this analysis.^23^

To assess the patients’ knowledge, the score was divided among four areas of knowledge, as it is in the original questionnaire. Area A referred to general knowledge, etiology, symptoms and blood tests; area B to immunogenic testing (B27) and inheritance; area C to drug treatment and physiotherapy; and area D to joint protection and energy conservation. There were low scores, but in three of the four areas, the average score was greater than 50% of the score for the total area. The exception was area B, which had a mean of 0.97 ± 0.85, with a maximum score for the area of 3 points. In the United Kingdom, the same area averaged 2.63 ± 0.52.^5^

During application of the questionnaire, it was observed that the patients who correctly answered that the antigen related to AS remembered they had already done this test for the diagnosis, but did not know that the antigen related to the disease. A small proportion correctly answered questions about genetic inheritance, but it was found that this question gave rise to the greatest number of doubts among the patients. Most of the patients believed that children had very high chances of also presenting the disease, and marked this alternative as correct. This area was the only one to present zero scores, and it was associated with a small range of values from zero to three. These were the most difficult questions in the questionnaire, and they asked for information that patients rarely receive, either from doctors or from physiotherapists. The average percentage of correct answers in this area was therefore very small: only 32.2% ± 28.3. The other areas showed average percentages of correct answers greater than 60%.

Area of knowledge A included general knowledge questions, etiology, symptoms and blood tests. The average percentage found was 64.6% ± 21.3, and the mean score was 5.17 ± 1.70, with a range between 2 and 8. In the British survey, the mean was 7.23 ± 0.73, and the minimum score was 5.

Area C included questions about drug treatment and physiotherapy. Most of the patients included in this study had already undergone physiotherapy or had participated in other previous studies on physical activity in relation to AS. As a consequence, the majority of the patients correctly answered the questions about the most appropriate type of physical activity for AS. We also obtained 100% correct answers to the question about the importance of exercise. In the British survey, the mean score was 8.81 ± 0.54, with a minimum score of 7. In France, 64% of the patients achieved 100% in this area, which was the highest percentage of patients who responded correctly, thus also showing a high level of knowledge in this area. Correlations between this area and other questionnaires were found.

Finally, for area D, which included issues relating to joint protection and energy conservation, the average score was 3.43 ± 1.04, with a minimum of 1 and maximum of 5. The average percentage of correct answers was 68.7% ± 20.8. In the British survey, the mean score was 4.74 ± 0.57. The average number of correct answers for this area of knowledge was reasonable, although a higher level of knowledge had been expected. Guidance of this nature is given both by physicians and by physiotherapists. In clinical practice, it was commonly observed that before receiving guidance on joint protection, patients had already adopted careful usage, given that after perceiving that certain ways of doing their daily and work activities led to increased or decreased pain, they began to perform these activities in different ways. Thus, the findings on the relationship between this area of knowledge and other questionnaires could be explained, in which we observed negative correlations with the domains of “pain”, “vitality” and “emotional aspects” of the SF-36 questionnaire. We could conclude that patients with lower scores in these domains, which means greater impairment, had higher levels of knowledge in this area.

In this study, and in the UK study, one important factor that needs to be borne in mind was that in order to answer each question, the patients considered what was most important in their experience with the disease and what was being asked about what they believed was the correct answer. For example, in question 2, there are two correct alternatives: “It is an inflammation in the joints of the spine” and “In some cases, the first complaint may not be in the lumbar region.” Many patients believed that the alternative “Worsening in the cold weather” was also correct, since, although this is not a rule, most of them felt that their symptoms worsened in cold weather. Furthermore, patients with recent diagnoses commonly thought that “No drug is able to control the disease,” was the correct alternative in Question 6, because once again, this correlated with the experience that they were going through with the medication. In question 7, patients with mild occurrences considered that the correct alternative was “Ankylosing spondylitis does not interfere in their work and physical activity,” despite knowing that there are other levels of involvement in the disease. Finally, in question 11, one patient did not consider “weight” to be the alternative for a type of physical activity indicated in cases of AS, since his/her experience with muscle strengthening had led to worsening of the symptoms.

The scores from the questionnaire assesed here cannot be compared with any other tool for assessing knowledge of AS among patients with this disease because no other instruments have been translated into Portuguese for this purpose.

The translation of this questionnaire makes clear the individual need of education about the disease, facilitating the evaluation and guidance of what should be done to each patient, optimizing the conduct regarding education. Other studies, however, should be performed in order to verify the effectiveness of the evaluation, comparing the scores before and after education sessions on the disease.

## CONCLUSION

The Brazilian version of the questionnaire “Ankylosing Spondylitis: What do you know?” was created. It is reproducible and correlates with education level, ethnicity and the SF-36 domains “social aspects” and “emotional aspects”.
